# Supplementary material for: TRPM2 ion channels steer neutrophils towards a source of hydrogen peroxide
Source: Sci Rep. 2021 Apr 29;11:9339. doi: 10.1038/s41598-021-88224-5 (PMC8085234; doi:10.1038/s41598-021-88224-5)
Supplement: Supplementary file 2 — Supplementary Video 1. [file 41598_2021_88224_MOESM2_ESM.pptx]

## Slide 1
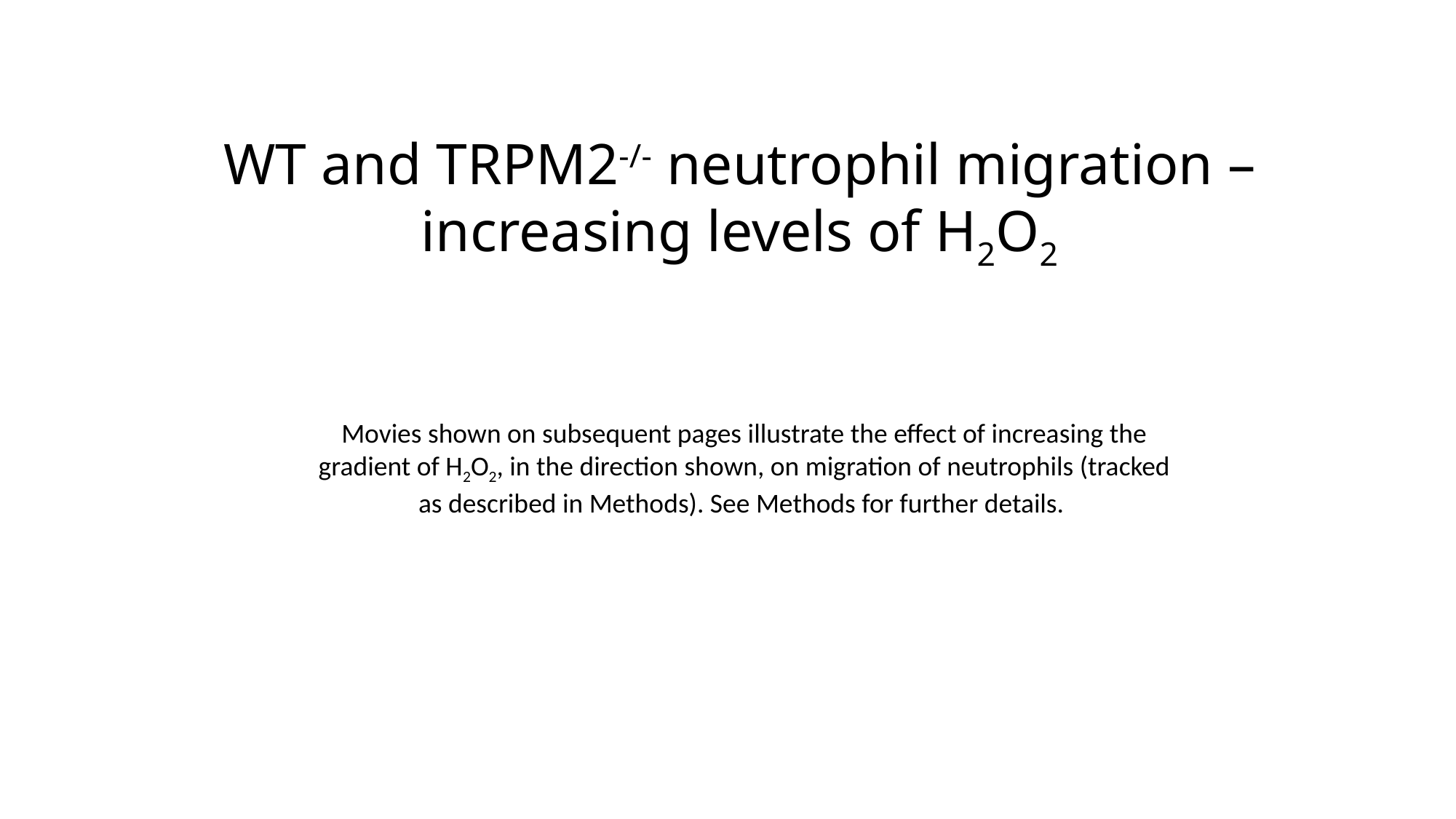

# WT and TRPM2-/- neutrophil migration – increasing levels of H2O2
Movies shown on subsequent pages illustrate the effect of increasing the gradient of H2O2, in the direction shown, on migration of neutrophils (tracked as described in Methods). See Methods for further details.

## Slide 2
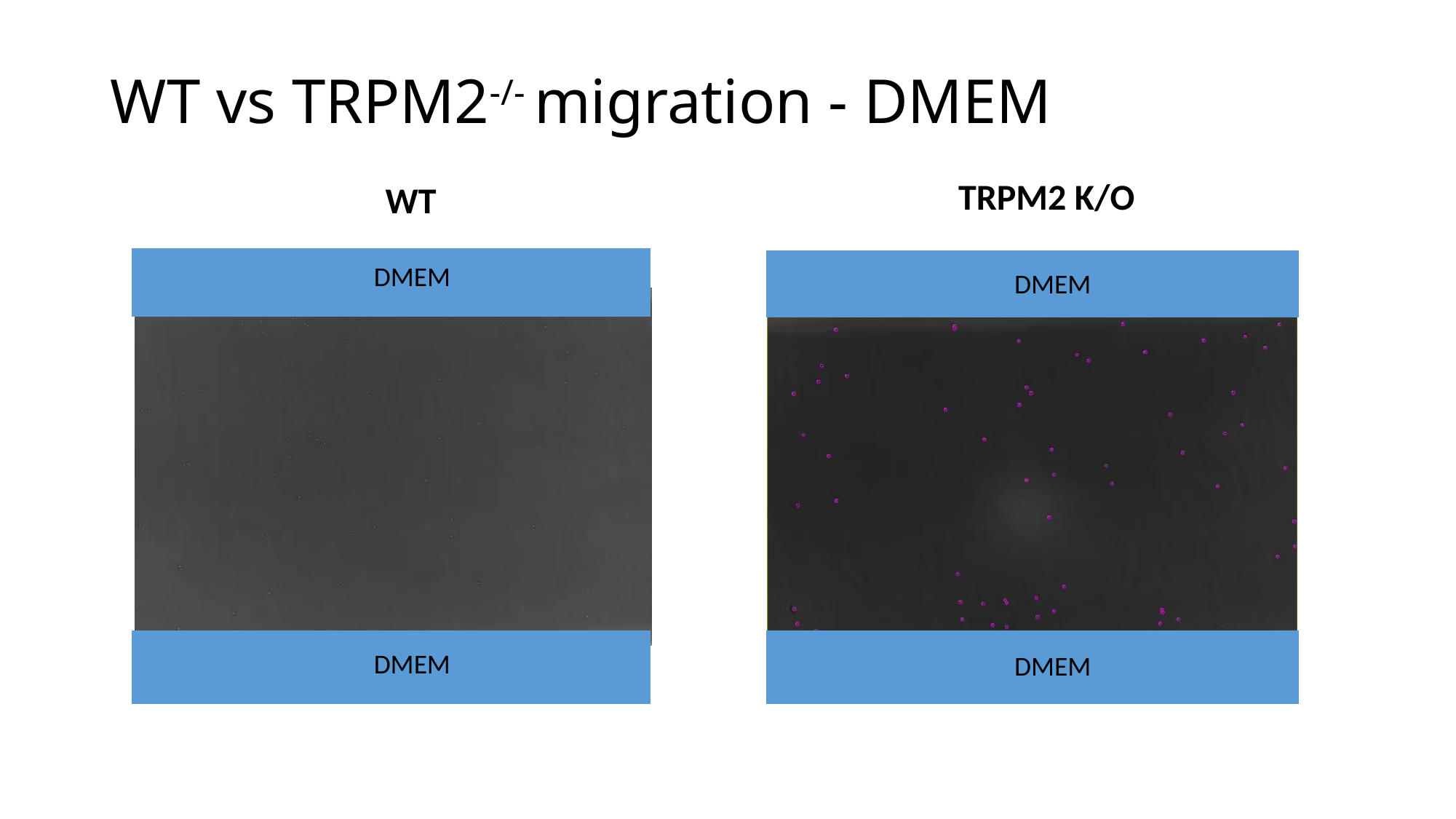

# WT vs TRPM2-/- migration - DMEM
TRPM2 K/O
WT
DMEM
DMEM
DMEM
DMEM

## Slide 3
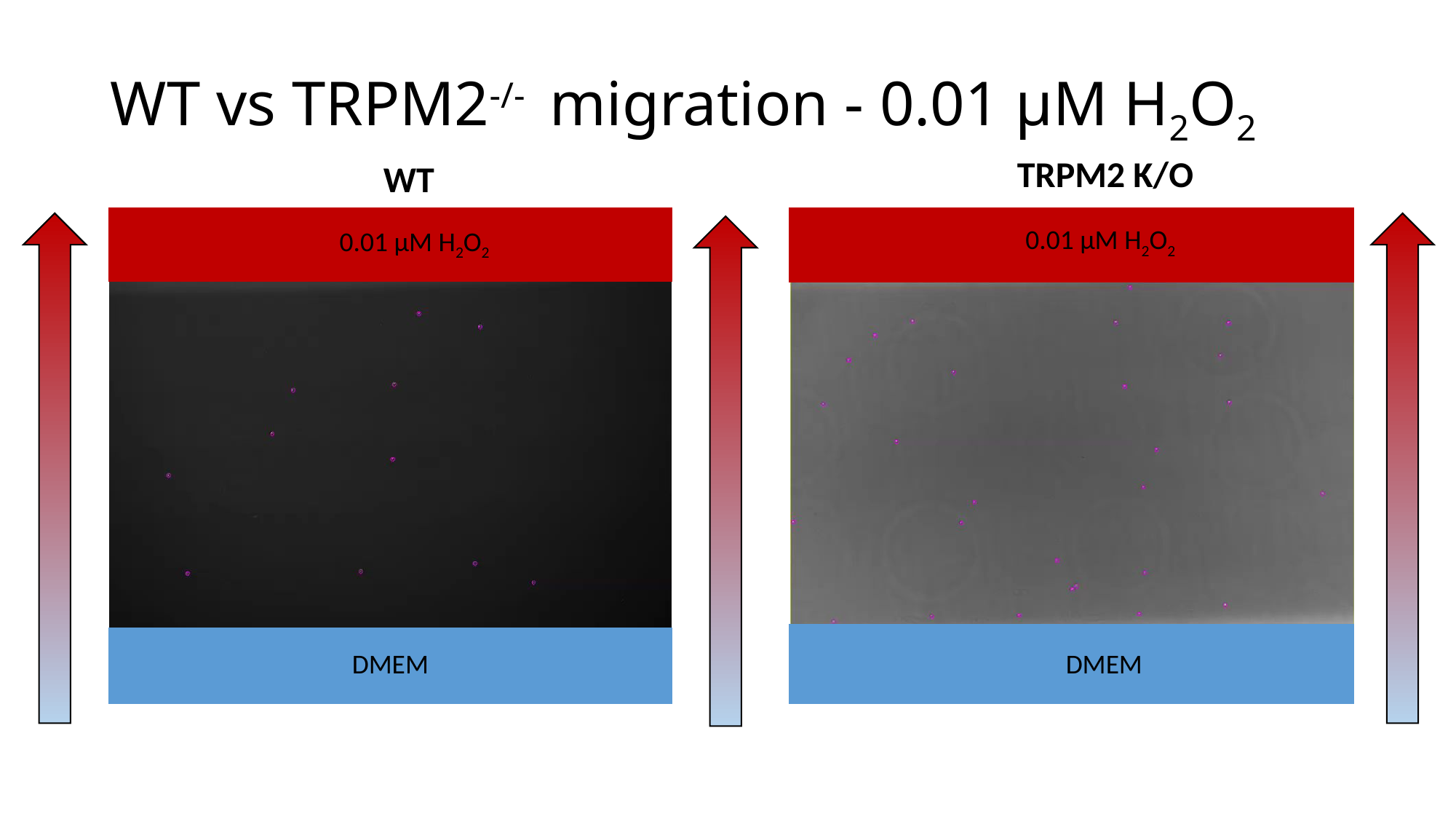

# WT vs TRPM2-/- migration - 0.01 µM H2O2
TRPM2 K/O
WT
0.01 µM H2O2
0.01 µM H2O2
DMEM
DMEM

## Slide 4
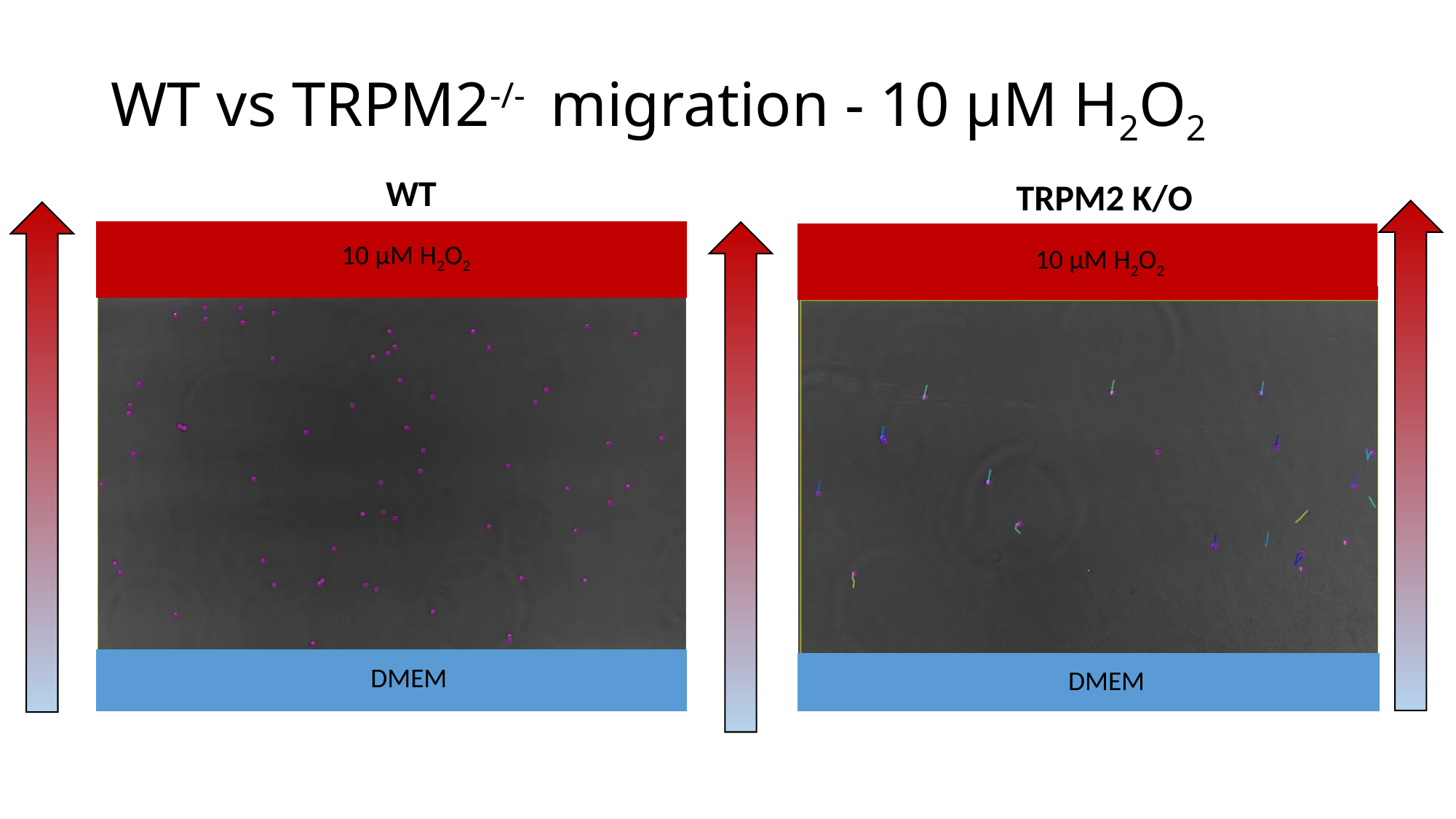

# WT vs TRPM2-/- migration - 10 µM H2O2
WT
TRPM2 K/O
10 µM H2O2
10 µM H2O2
DMEM
DMEM

## Slide 5
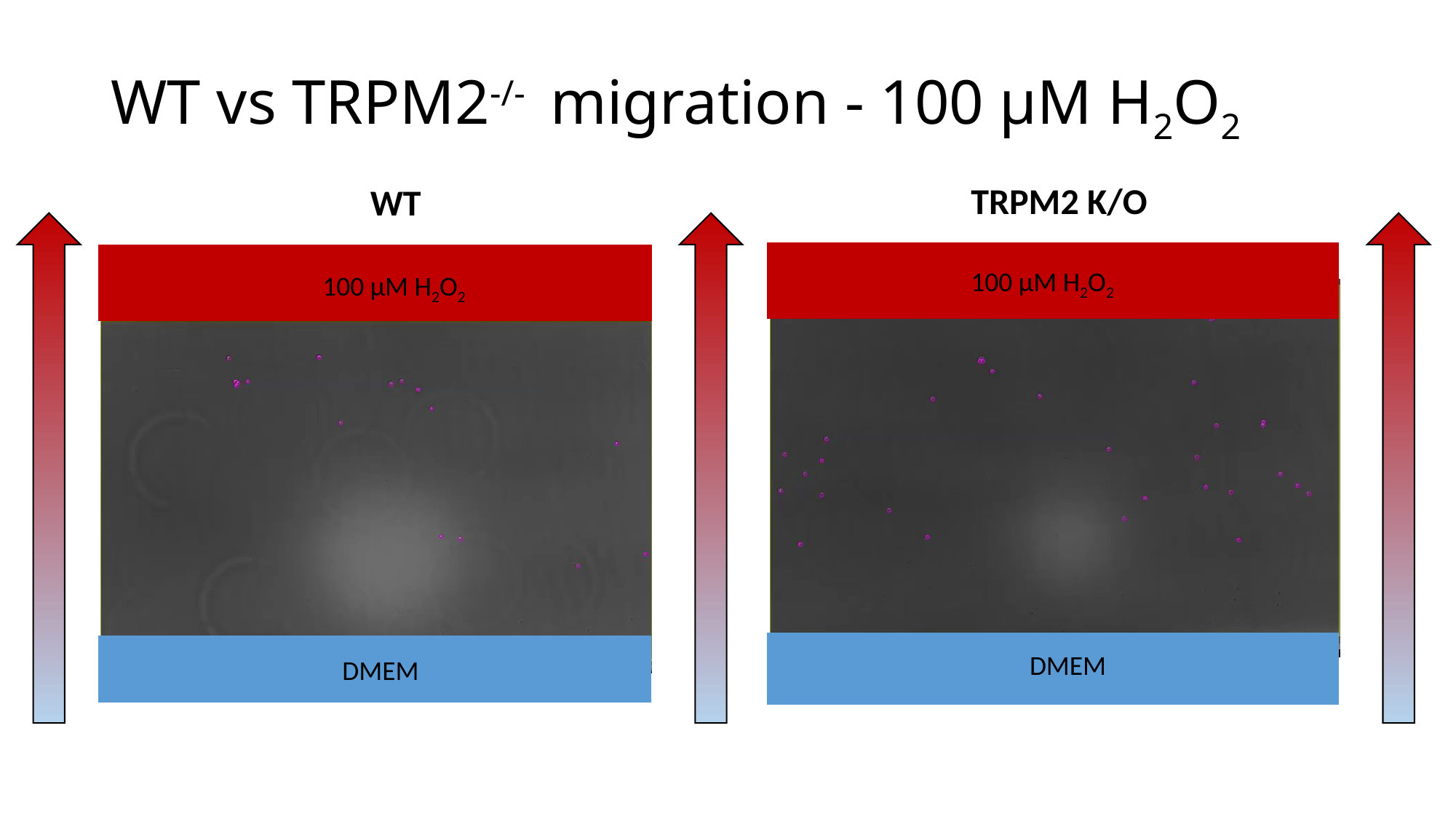

# WT vs TRPM2-/- migration - 100 µM H2O2
TRPM2 K/O
WT
100 µM H2O2
100 µM H2O2
DMEM
DMEM
