# Supplementary material for: TRPM2 ion channels steer neutrophils towards a source of hydrogen peroxide
Source: Sci Rep. 2021 Apr 29;11:9339. doi: 10.1038/s41598-021-88224-5 (PMC8085234; doi:10.1038/s41598-021-88224-5)
Supplement: Supplementary file 3 — Supplementary Video 2. [file 41598_2021_88224_MOESM3_ESM.pptx]

## Slide 1
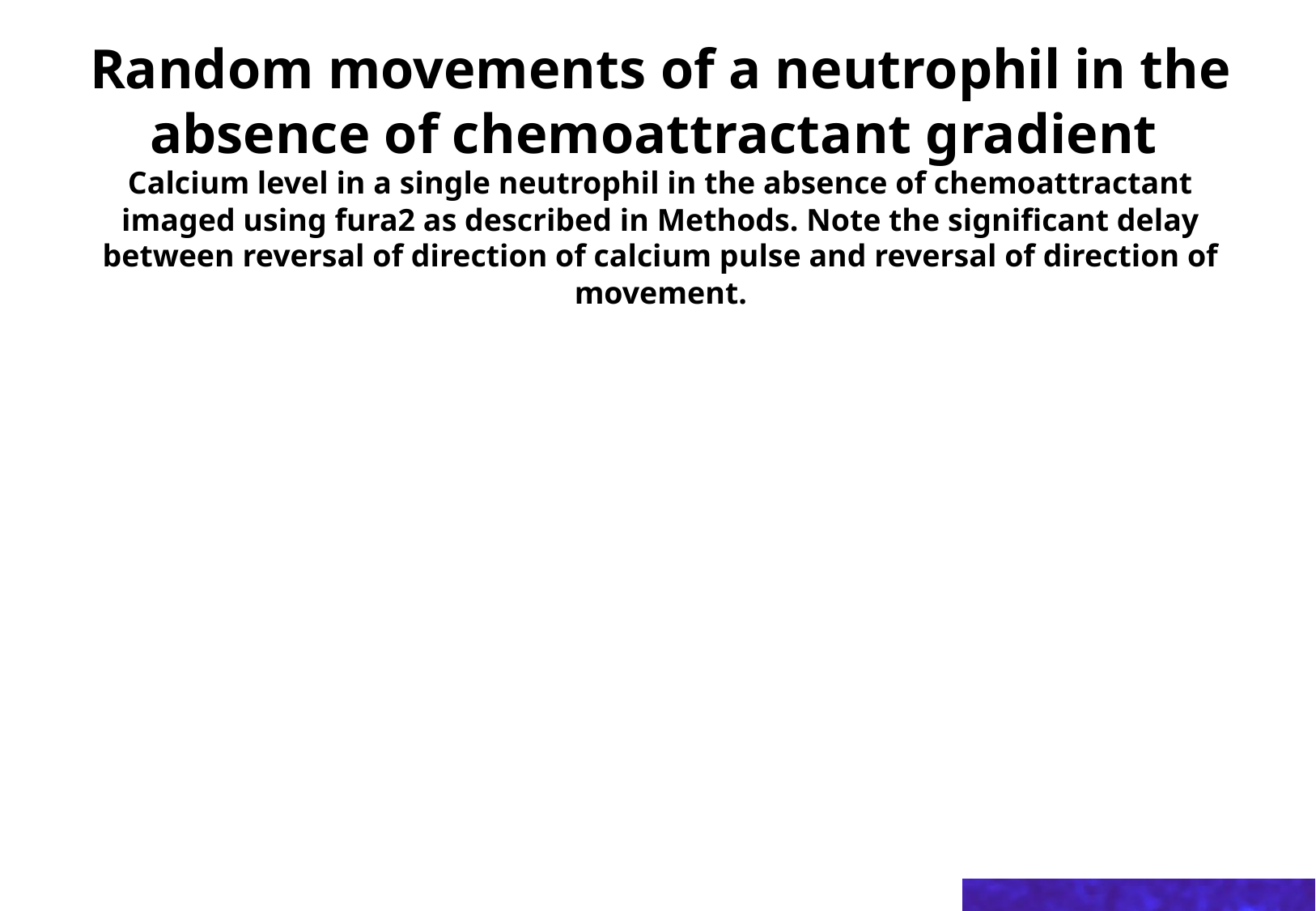

Random movements of a neutrophil in the absence of chemoattractant gradient
Calcium level in a single neutrophil in the absence of chemoattractant imaged using fura2 as described in Methods. Note the significant delay between reversal of direction of calcium pulse and reversal of direction of movement.
